# Association of Diet Quality and Vegetable Variety with the Risk of Cognitive Decline in Chinese Older Adults

**DOI:** 10.3390/nu11071666

**Published:** 2019-07-20

**Authors:** Yi-Chun Chou, Meei-Shyuan Lee, Jeng-Min Chiou, Ta-Fu Chen, Yen-Ching Chen, Jen-Hau Chen

**Affiliations:** 1 Department of Geriatrics and Gerontology, National Taiwan University Hospital, No.1, Changde Street, Taipei 10048, Taiwan; 2 Institute of Epidemiology and Preventive Medicine, College of Public Health, National Taiwan University, No. 17 Xu-Zhou Road, Taipei 10055, Taiwan; 3 Department of Public Health, School of Public Health, National Defense Medical College, No. 161, Sec. 6, Minquan E. Road, Neihu District, Taipei 11490, Taiwan; 4 Institute of Statistical Science, Academia Sinica, 128 Academia Road, Section 2, Nankang District, Taipei 11529, Taiwan; 5 Department of Neurology, National Taiwan University Hospital, No. 1, Changde Street, Taipei 10048, Taiwan; 6 Department of Public Health, College of Public Health, National Taiwan University, No. 17, Xu-Zhou Road, Taipei 10055, Taiwan

**Keywords:** diet quality, vegetable variety, cognitive decline, older adults

## Abstract

Diet quality plays an important role in dementia prevention. It remains unclear how the joint effect of vegetable variety and diet quality affects cognition. This study aimed to explore the association of diet quality and vegetable variety with cognitive decline in older adults. This prospective cohort study (2011–2015) included 436 community-dwelling elders in Taipei. Diet quality, assessed by the modified Alternative Healthy Eating Index (mAHEI), was computed from a food frequency questionnaire at baseline (2011–2013). Vegetable variety indicated the number of different vegetable groups, adjusted for vegetable quantity. Multivariable linear and logistic regression models were used to explore the association of diet quality and vegetable variety with the decline of global and domain-specific cognition over two years. Our findings suggest that high diet quality (the highest tertile of mAHEI) was associated with a lower risk of both global cognitive decline (adjusted odds ratio (AOR) = 0.54, confidence interval (CI) = 0.31–0.95) and decline of attention domain (AOR = 0.56, CI = 0.32–0.99) compared with low diet quality. In elders with high vegetable variety, high diet quality was associated with a lower risk of global cognitive decline (AOR = 0.49, CI = 0.26–0.95). We therefore concluded that high diet quality along with diverse vegetable intake was associated with a lower risk of cognitive decline in older adults.

## 1. Introduction

As the global population ages rapidly, dementia has become an essential public health concern as it places a substantial burden on society as a whole. Mild cognitive impairment (MCI), characterized by objective cognitive decline in older adults but with the preservation of daily functioning, is viewed as a transition stage between normal cognitive aging and dementia [1,2]. Dementia is characterized by cognitive decline in at least one cognitive domain that interferes with the independence of daily activities. Among them, Alzheimer’s disease (AD) is the leading cause [1,3]. The estimated number of people with dementia worldwide doubles every 20 years, and the incident dementia cases in Asia account for the majority (49%) of it [4]. A large survey in Taiwan (2011–2013) reported that the age–gender-adjusted prevalence was 18.78% for MCI and 8.13% for all-cause dementia in older adults (age ≥ 65 years) [5]. Currently, some medications are used for symptomatic treatments of AD or mixed dementia. However, the disease pathogenesis cannot be altered, and will eventually lead to death [3,6]. This reflects the importance of predicting the risk of cognitive decline in the pre-clinical phase and understanding the factors contributing to individual differences of cognitive aging.

The causes for late-onset AD (LOAD) are multifactorial. Old age, family history of AD, and carrying the *APOE* e4 allele (an important genetic marker for both early and late-onset AD) are important non-modifiable risk factors [3,7,8]. However, several modifiable lifestyle factors, including regular physical activities, cognitive training, and dietary factors (e.g., n-3 fatty acid, vegetable intake, and Mediterranean Diet pattern) are known to be associated with cognitive decline [9]. These environmental factors may influence brain health and cognition by modulating epigenetic changes [10]. Older adults’ nutritional status is often compromised. They tend to reduce food intake due to decreased activities and basal metabolic rate, but the demands for specific nutrients remain unchanged or increased [11]. Therefore, a good diet quality is important for older adults.

Dietary intake is composed of several food items and nutrients that may interact with each other. In the past decades, dietary pattern has emerged as an important approach to assess the overall diet intake and its association with different outcomes (e.g., coronary heart disease and cancer) [12,13]. Predefined diet quality indexes are one of the dietary pattern-based strategies. This a priori (or hypothesis-oriented) approach constructs dietary pattern scores based on current nutrition guidelines or recommendations [14]. However, the variety within food groups is also an important element of healthy eating, which is usually not considered in predefined diet quality indexes [15]. Dietary guidelines recommend choosing a variety of foods within all food groups, especially for vegetable subgroups and proteinaceous foods [16,17,18]. Vegetables are a heterogenous food group rich in B vitamins and antioxidants (vitamins A, C, and E), which may protect against cognitive decline [19]. Previous studies in both Eastern and Western countries have shown consistent evidence that vegetable intake is beneficial to cognitive function [9,20,21,22]. Therefore, assessing the joint effect of vegetable variety and diet quality may provide a useful tool to evaluate the overall nutritional status in older adults.

Previous studies on diet quality and cognitive impairment in older adults have been inconsistent. Cross-sectional studies showed that higher diet quality (assessed by the Healthy Diet Indicator, HDI) was associated with the risk of cognitive impairment [23,24]. In contrast, cohort studies found no association between diet quality (as assessed by Healthy Eating Index (HEI)-2005, Alternate Healthy Eating Index (AHEI)-2010, Canadian Healthy Eating Index (C-HEI), or HDI) and global cognitive decline [25,26,27,28]. The inconsistency may be attributable to different geographic regions, dietary habits, assessment tools for diet quality and cognition, and covariates adjustment.

Most studies focused on western populations (e.g., Italy [24], Sweden [27], United States [25,28], and Canada [26]). Our previous study identified specific dietary patterns for older Chinese adults via an a posteriori (or data-driven) approach [22]. However, these dietary patterns may not be easily compared with other studies because food items, cooking style, and diet habit are quite different across geographical regions. In addition, it is unclear how predefined diet quality and vegetable variety—both of which reflect adherence to the recommendations of nutrition guidelines—affect cognitive function in this old population. Therefore, this study aimed to explore the association of diet quality and vegetable variety with the decline of global and domain-specific cognition in community-dwelling older Chinese adults, and followed up on their cognition after two years. Stratified analyses by important covariates (i.e., vegetable variety, age groups, sex, and *APOE* e4 status) were performed to explore how they affected the association above.

## 2. Materials and Methods

### 2.1. Study Population

This study is part of an ongoing prospective cohort study, the Taiwan Initiative for Geriatric Epidemiological Research (TIGER), which recruited community-dwelling older adults in Taiwan. A total of 605 participants aged 65 years or older were recruited from the annual senior health checkup program at National Taiwan University Hospital (NTUH) between August 2011 and July 2013. Participants with the following conditions were excluded (*n* = 97): history of stroke (*n* = 10), brain tumor (≥3 cm) found by magnetic resonance imaging (*n* = 2), history of head trauma (*n* = 4), baseline Montreal Cognitive Assessment—Taiwanese version (MoCA-T) score ≤ 21 (*n* = 56), use of medications for treating AD (*n* = 2), incomplete data of food frequency questionnaire (FFQ, *n* = 5), and unreasonable total energy intakes (<650 kcal/day [29], *n* = 1) or physical activities (>10,000 MET-min/week, *n* = 1). Vegetarians (*n* = 15) were excluded because the FFQ was not designed for this subpopulation. One participant with a high point loss over 2 years in the MoCA-T score (≥12, *n* = 1) was also excluded. After exclusion, 72 out of 508 older adults were lost to follow-up (participation rate = 86%). A total of 436 older adults were included for statistical analysis. The study protocol, informed consent, questionnaires, and application forms were approved by the Research Ethics Committee at NTUH. Each study participant provided written informed consent before enrollment.

### 2.2. Assessment of Diet, Diet Quality, and Vegetable Variety

Dietary intake in the year prior to the baseline was recorded by a 44-item semi-quantitative FFQ. This questionnaire was derived from a validated 64-item FFQ for the Taiwanese population [30]. The intake frequency of each food item was converted into a standard daily serving based on Dietary Guidelines for Americans (DGA) and Food Patterns Equivalents database for generating the modified Alternative Healthy Eating Index (mAHEI) [31]. Total energy intake was estimated according to Taiwan’s Food and Drug Administration database [32].

Diet quality was evaluated by the mAHEI [33]. Seven food components (i.e., fruits, vegetables, soy protein, fish/(meat + eggs), whole grain, fried foods, and alcohol) were included in the mAHEI (Supplementary Table S1). Nuts were excluded from the component of “nuts and soy protein” because nuts are not commonly consumed by older Chinese adults. For each component, a proportional score was computed based on the minimum (0) and maximum (10) criteria. Scores obtained from each of the seven components were summed up into a composite score, ranging from 0 to 70 (Supplementary Table S2). After sorting by the mAHEI score, study participants were tertiled into low (T1), moderate (T2), and high (T3) diet quality. Higher scores indicated higher diet quality and better adherence to dietary recommendations (e.g., higher intake of fruits and vegetables compared to their counterparts).

Vegetable variety was derived from the diet diversity score (DDS) [34]. In order to reflect our diet habit, the classification of vegetable subgroups in the DGA was modified to assess vegetable variety, which included five vegetable subgroups: (1) spinach and broccoli, (2) other dark-green vegetables, (3) red and orange vegetables, (4) total starchy vegetables, and (5) other vegetables [16]. For each vegetable subgroup, “1” indicated the intake of one cup-equivalent (c-eq) per week, and “0” indicated a lesser amount. The total score of vegetable variety ranged from 0 to 5. The higher the score, the greater the vegetable variety.

### 2.3. Assessment of Cognition

Global and domain-specific cognition were evaluated using various neuropsychological tests. The MoCA-T was used for assessing global cognition in Taiwanese individuals, with the cutoff points slightly different from other ethnic groups (dementia: ≤21, MCI: 22–23, normal cognition: ≥24) [35]. For domain-specific cognition, eleven tests detailed below were performed and then grouped into four domains. Wechsler Memory Scale-Third edition (WMS-III) was used to assess logical memory (theme I and II; and recall I and II) and attention domains (digit span-forward and backward) [36]. The tests for evaluating verbal fluency included naming fish, vegetables, and fruit within one minute. The tests for assessing executive function included trail-making tests A and B.

These tests were performed at baseline as well as at the 2-year follow-up. For each domain-specific cognitive variable, a Z-score was calculated by dividing the difference between the observed value and baseline mean by the baseline standard deviation of the cohort [37]. Composite Z-scores for each of the four domains were generated by averaging cognitive variables in the same domains. Changes of global cognition and four cognitive domains over two years were then tertiled (T1, T2, and T3); cognitive decline was defined as the lowest tertile (T1) of the cognitive change, and the higher tertiles (T2 + T3) indicated normal cognition.

### 2.4. Covariates

A self-reported questionnaire was administered to obtain information on demography (e.g., age, sex, years of education, and annual disposable income), medical history, and supplement use. Weight and height were measured to calculate body mass index (weight in kilograms divided by height in meters squared). Gait speed was measured as the time in seconds for walking 2.4 m. Physical activity in the past week was assessed by a short version of the International Physical Activity Questionnaire (IPAQ) [38]. A 20-item version of the Center for Epidemiologic Studies Depression (CES-D) Scale with a total score of 60 was used to assess depressive symptomatology [39]. Presence of depressive symptoms was defined as CES-D ≥ 16 [39], use of antidepressants, or self-reported depression diagnosis. Hypertension was defined as high blood pressure (systolic blood pressure ≥ 140 mmHg or diastolic blood pressure ≥ 90 mmHg), use of anti-hypertensives, or self-reported hypertension history. Diabetes was defined as blood fasting glucose ≥ 126 ng/dL, use of oral hypoglycemia agents/insulin, or self-reported diabetes history.

### 2.5. Laboratory Assay

Blood fasting glucose was included in the package of the senior health checkup program. Genomic DNA was extracted from buffy coat using the QuickGene-Mini 80 system (Fujifilm, Tokyo, Japan). *APOE* e4 status was determined by two single-nucleotide polymorphisms, rs429358 and rs7412, which were genotyped by TaqMan Genomic Assays using the ABI 7900HT fast real-time PCR system (Applied Biosystems Inc., Foster City, CA, USA).

### 2.6. Statistical Analysis

Due to the issue of moderate correlation between vegetable variety score and vegetable intake (*r* = 0.56, *p* < 0.0001), the vegetable variety score was regressed on the total amount of vegetable intake to resolve the concern of collinearity [40]. The residuals of vegetable variety score (quantity-adjusted vegetable variety score) were then generated to explore the association of diet quality and quantity-adjusted vegetable variety score with cognitive decline.

One-way analysis of variance (ANOVA) (for continuous variables) and chi-square test (for categorical variables) were used to compare the distribution of variables across tertiles of the diet quality score and quantity-adjusted vegetable variety score, respectively. Multivariable linear regression was used to estimate the change of global and domain-specific cognition for one tertile increase of diet quality score or quantity-adjusted vegetable variety score. Multivariable logistic regression was used to estimate adjusted odds ratio (AOR) and 95% confidence interval (CI) for the risk of cognitive decline in global and domain-specific cognition over 2 years comparing high (T3) and moderate (T2) with low (T1) diet quality or quantity-adjusted vegetable variety, respectively. All models were adjusted for variables with biological importance: age, sex, years of education, *APOE* e4 status, baseline cognition (global or domain-specific), total energy intake, and depressive symptoms. Variables identified by stepwise selection (SLENTRY = 0.10) and found to be significantly associated with the outcome variables in the multivariable analyses were further adjusted in the models. Stratified analyses were performed to evaluate how the association of diet quality and the risk of cognitive decline were affected by some important covariates (i.e., quantity-adjusted vegetable variety score T1 vs. T2 + T3), age groups (65–74 vs. ≥75), sex, and *APOE* e4 status. We then tested the interactions between diet quality and important covariates by comparing models with terms of main effects and interaction terms to models with main effects only (*p_interaction_* < 0.10 indicated the presence of an interaction). A two-sided *p* < 0.05 was considered statistically significant. Data analyses were performed using SAS version 9.4 (SAS Institute Inc., Cary, NC, USA).

## 3. Results

### 3.1. Characteristics of the Study Population

Among 436 participants included for statistical analyses, the mAHEI score (a measure of diet quality) ranged from 17.2 (poor diet quality) to 57.3 (good diet quality), with a mean of 36.5. Vegetable variety, age, physical activities, gait speed, annual disposable income, and the presence of hypertension differed significantly across the tertiles of diet quality (Table 1). The mean diet quality score and annual disposable income differed significantly across the tertiles of quantity-adjusted vegetable variety score (Supplementary Table S3).

### 3.2. Association between Diet Quality, Quantity-Adjusted Vegetable Variety, and Risk of Cognitive Decline

High diet quality (T3) was associated with a lower risk of global cognitive decline (AOR = 0.54, 95% CI = 0.31–0.95, *p_trend_* = 0.03, Table 2) compared with low diet quality (T1). For cognitive domains, high diet quality (T3) was associated with a lower risk of decline of attention domain (AOR = 0.56, 95% CI = 0.32–0.99, *p_trend_* = 0.049). No significant association was found between quantity-adjusted vegetable variety and the risk of cognitive decline (Table 3). The results of multivariable linear regression showed similar findings (Table 2 and Table 3).

### 3.3. Interactions between Diet Quality and Important Covariates

**Figure 1:**
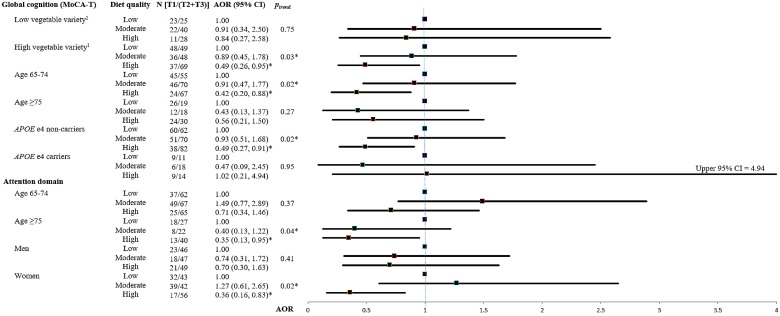
Forest plot of the association between diet quality and cognitive decline over 2 years stratified by important covariates. Cognitive decline was defined as the lowest tertile (T1) of the cognitive change over 2 years, and the higher tertiles (T2 + T3) indicated normal cognition. ^1^ Low vegetable variety was defined as the lowest tertile (T1) of the quantity-adjusted vegetable variety, and high vegetable variety was defined as the higher tertiles (T2 + T3). Multivariable models were adjusted for age, sex, years of education, *APOE* e4 status, the corresponding cognitive variable at baseline, total calories, depressive symptoms, and quantity-adjusted vegetable variety. Additional variables adjusted for different outcome variables included annual disposable income for global cognition. Abbreviations: MoCA-T, Montreal Cognitive Assessment—Taiwanese version; T, tertile; AOR, adjusted odds ratio; CI, confidence interval; *APOE,* apolipoprotein E. * *p_trend_* < 0.05

Stratified analyses by important covariates were performed for significant associations found between diet quality and cognitive decline in the aforementioned regression analyses. These covariates included quantity-adjusted vegetable variety (T1 vs. T2 + T3), age groups (65–74 vs. ≥75), sex, and *APOE* e4 status (Supplementary Table S4). The stratified analyses with significant findings are presented in the forest plot (Figure 1).

Significant interactions were found between *APOE* e4 status and diet quality in the risk of global cognition (*p_interaction_* = 0.05) and attention decline (*p_interaction_* = 0.06). However, no significant interaction was found in the remaining stratified analyses. After stratified analyses, significant findings were found in some subgroups, as below. For participants with high vegetable diversity (T2 + T3), high diet quality was associated with a lower risk of global cognitive decline (AOR = 0.49, 95% CI = 0.26–0.95, *p_trend_* = 0.03) compared with low diet quality. For other important covariates, high diet quality was associated with a lower risk of global cognitive decline (MoCA-T) in elders aged 65–74 (AOR = 0.42, 95% CI = 0.20–0.88, *p_trend_* = 0.02) and *APOE* e4 non-carriers (AOR = 0.49, 95% CI = 0.27–0.91, *p_trend_* = 0.02). For cognitive domains, high diet quality was associated with a lower risk of attention decline in elders aged ≥75 (AOR = 0.35, 95% CI = 0.13-0.95, *p_trend_* = 0.04) and women (AOR = 0.36, 95% CI = 0.16–0.83, *p_trend_* = 0.02).

## 4. Discussion

To the best of our knowledge, this is the first study exploring the association between diet quality and vegetable variety on cognition in older adults without dementia. We found that high diet quality (high intake of vegetables, fruits, whole grains, soy protein; higher intake of fish and seafood relative to meat, poultry, and eggs; moderate drinking; as well as low intake of deep-fried foods) was associated with a lower risk of both global cognitive decline (assessed by MoCA-T) and decline of attention domain (assessed by digit span-forward and backward) over 2 years. The association of high diet quality with reduced risk of global cognitive decline became stronger in elders with high vegetable diversity intake. Taken together, adherence to nutritional guidelines (meeting the recommendations of food groups and selecting diverse vegetable subgroups) reduced the risk of cognitive decline in older Chinese adults.

Previous studies found that diet quality was not associated with cognition [25,26,27,41]. This inconsistency may be attributable to the following reasons. First, the Chinese take in 10- to 40-fold more soy products (e.g., soymilk and tofu) than Western populations (e.g., United States and Western Europe) [42,43,44]. Isoflavones in soy products are known to improve cognition via their interactions with estrogen receptors in the hippocampus and prefrontal cortex [42,45]. Second, the Chinese tend to add oil while they sauté or stir-fry foods. The most commonly consumed oils in our study were olive oil (58%) and sunflower oil (17%). Olive oil which is rich in monounsaturated fatty acids (MUFAs) and natural antioxidants (e.g., polyphenols) has been associated with less cognitive decline [46,47]. In addition, cooking oil rich in vitamin E could enhance the absorption of vitamin E and other lipophilic nutrients, which are known to be beneficial to cognition [20]. Third, different construction criteria across diet quality indices (Supplementary Table S5) may also influence the association between diet quality and cognition. For example, except mAHEI, most of diet quality indices (e.g., AHEI-2010, HEI-2005, C-HEI, and HDI) gave more weights (36% to 63%) to the moderation components (e.g., sodium and total carbohydrates), which may understate the remaining components (e.g., vegetables, fruits, and whole grains) [27,48,49,50]. These reasons may explain previous null findings between diet quality index and cognition in older adults. Lastly, some studies enrolled participants from clinical trials and found that high diet quality showed neuroprotective effects in middle-aged adults [51,52]. However, their findings may be due to younger and highly selected participants (e.g., higher education level or lower disease severity in order to participate in the study), who tend to have less heterogeneity than older adults.

Diet quality may influence cognition via the protective effects of food items. For foods acting as antioxidants and free-radical scavengers, they could reduce oxidative damage, down-regulate inflammation, and strengthen the neurons’ antioxidant defense [53,54]. Vegetables, fruit, soy products, and whole grains are rich in antioxidant and phenolic compounds (e.g., flavonoids and lignans). They have shown a neuroprotective effect via ameliorating the effects of oxidative stress (OS) and inflammation [55]. Seafood and fish are rich in n-3 fatty acids, which are anti-thrombotic and are also known to maintain the integrity of neuronal membrane [19]. Furthermore, a high-quality diet may delay cognitive decline by modifying the risk of other vascular diseases (e.g., stroke and cardiovascular events), which are risk factors of cognitive impairment [33,56,57]. This may explain our finding that high-quality diet was associated with a lower risk of the impairment of attention domain, whose deficit is more prominent in vascular cognitive impairment than in AD [57]. These may support that high diet quality was associated with a lower risk of decline of global cognition and attention domain in our study.

Our study did not find an association between quantity-adjusted vegetable variety and cognitive decline. However, the only cross-sectional study showed that higher vegetable variety was associated with better executive function and attention in both middle-aged and older adults [58]. It is possible that the neuroprotective effect differs across vegetable subgroups (e.g., dark-green, red and orange vegetables, etc.). However, the variety score we adopted may not be able to reflect the differential protective effects because equal weight was assigned to all vegetable subgroups. Green leafy vegetables (e.g., spinach), which are rich in phenolic compounds, lutein, vitamin E, and folate, have been shown to protect against cognitive decline [21,59,60]. However, the protective effect was not found in yellow vegetables [21,60]. Furthermore, the consumption of vegetable subgroups differs across ethnic groups and geographical areas. For example, older adults in our study commonly consume dark-green vegetables (mean: 5.1 c-eq/week); in contrast, older American adults tend to consume more (1) starchy vegetables and (2) vegetables other than dark-green, red and orange, starchy vegetables, and legumes defined by DGA [16]. Future studies are warranted to investigate the effect of different vegetable patterns or variety score considering different weighting according to dietary recommendations to clarify their relation with cognition.

Although increased vegetable variety alone was not associated with cognitive function in our study, the positive association of high diet quality with reduced risk of global cognitive decline became stronger in elders with high vegetable diversity intake. Vegetables are low-energy-density foods. Unlike other non-vegetable foods, increasing vegetable variety may lead to the substitution of higher fat foods, which not only improves the overall diet profile but also prevents the risk of being obese [61,62]. The combination of high diet quality (between-group variety) and high vegetable variety (within-group variety) promotes more nutrient adequacy than the effect of each alone [63]. In addition, we found a significant association between diet quality and the attention domain in women, which may be the result of their younger age compared to men (71.3 vs. 72.8, *p* < 0.0001). Lastly, *APOE* gene involved in lipoprotein metabolism is known to modify the effectiveness of diet intervention and AD [64]. This may also explain the significant interaction between *APOE* e4 status and diet quality in cognitive function observed in our study.

Our study has several strengths. First, because of the high correlation between vegetable variety and diet quality, we used a residuals method to avoid the collinearity issue while exploring the association of vegetable variety, diet quality, and cognition. Second, this study had information of several important covariates (e.g., *APOE* e4 status, depressive symptoms, socioeconomic status and lifestyle factors), which allowed us to control for them in the multivariable regression models. Third, in order to identify cognitive impairment at the pre-clinical phase, our study used MoCA-T, which is more sensitive than MMSE, to detect mild cognitive impairment or subtle change in global cognition in this non-demented elderly population [35]. We also collected domain-specific cognition to provide more details of the outcome variables than previous studies. Lastly, we excluded participants with dementia at baseline in order to reduce the impact of impaired memory on the validity of the FFQ [65]. This also allowed us to focus on non-demented older adults for early prevention.

On the other hand, our study has some limitations. First, the FFQ adopted by this study was relatively short and therefore unable to estimate the amount of nutrient intake or moderation (i.e., sodium or empty calories), which were required components in some diet quality indexes. Second, the classification of vegetable subgroups (e.g., spinach and broccoli, and other dark-green vegetables) modified from DGA may not adequately capture the high vegetable diversity in Taiwan. Third, our participants were recruited from a senior health checkup program in Taipei City, who tended to have higher education level, annual disposable income, and be more health-conscious than the general population. However, as the follow-up time increased, the health status of these older adults gradually declined and became similar to that of the general population.

## 5. Conclusions

Our findings suggest that high diet quality along with diverse vegetable intake is associated with a lower risk of cognitive decline in the older adults. Further studies with longer follow-up, different geographical areas, and larger sample size are warranted to confirm our findings.

## Figures and Tables

**Table 1:** The characteristics of the study population by tertiles of diet quality at baseline (2011–2013).

Variables	Diet Quality (mAHEI)				*p*-Value
	Overall	Low (T1)	Moderate (T2)	High (T3)	
	(*n =* 436)	(*n =* 145)	(*n =* 146)	(*n =* 145)	
	Mean, SD				
mAHEI	36.5, 7.6	28.1, 4.1	36.6, 2.0	44.9, 3.6	<0.0001
Vegetable variety ^1^	3.4, 1.0	3.1, 1.1	3.3, 0.9	3.9, 0.8	<0.0001
Age (years)	72.5, 5.2	72.8, 5.2	71.4, 4.7	73.1, 5.6	0.01
Years of education (years)	14.0, 3.4	13.5, 3.4	14.1, 3.3	14.4, 3.6	0.08
BMI (kg/m^2^)	23.8, 2.9	24.1, 2.8	23.9, 3.1	23.5, 2.9	0.26
Physical activity (MET-min/week)	1747.5, 1444.0	1494.5, 1418.4	1821.7, 1506.7	1925.9, 1377.6	0.03
Gait speed (m/s)	0.8, 0.2	0.8, 0.2	0.9, 0.2	0.9, 0.3	0.04
Total energy intake (kcal/day)	1683.0, 395.1	1632.8, 415.6	1684.5, 377.4	1731.6, 387.9	0.10
MoCA-T	27.0, 2.1	27.0, 2.1	27.1, 2.1	27.0, 2.2	0.86
	N (%)				
Women	231 (53)	75 (52)	81 (55)	75 (52)	0.76
Annual disposable income (TWD per year)					<0.0001
<300 K	68 (16)	30 (23)	18 (13)	20 (14)	
300–800 K	106 (26)	47 (35)	31 (22)	28 (20)	
800–1000 K	61 (15)	18 (13)	10 (7)	33 (24)	
>1000 K	177 (43)	38 (29)	81 (58)	58 (42)	
Supplement use	346 (79)	113 (78)	124 (85)	109 (75)	0.11
Depressive symptoms ^2^	43 (10)	18 (12)	14 (10)	11 (8)	0.38
Hypertension	280 (65)	106 (74)	89 (61)	85 (60)	0.02
Diabetes mellitus	68 (16)	24 (17)	27 (18)	17 (12)	0.26
*APOE* e4 carriers	67 (16)	20 (14)	24 (17)	23 (16)	0.83

^1^ Vegetable variety was defined as the number of five vegetable groups consumed at least one cup equivalent per week (score range: 0–5). ^2^ Depressive symptoms (yes/no) were defined as at least one of the following three factors: CES-D scores ≥ 16, use of antidepressants, or self-report diagnosis of depression. Abbreviations: mAHEI, modified Alternative Healthy Eating Index; T, tertile; SD, standard deviation; BMI, body mass index; MET, metabolic equivalent of task; MoCA-T, Montreal Cognitive Assessment—Taiwanese version; TWD, Taiwan dollar; *APOE*, apolipoprotein E; CES-D, Center for Epidemiologic Studies Depression. Numbers in bold indicate significant findings (*p* < 0.05).

**Table 2:** Association between diet quality (mAHEI) and cognitive decline over 2 years.

Cognitive Function	Diet Quality	β (95% CI) ^1^^,^^3^	N (Decliners/Non-Decliners)	AOR (95% CI) ^2,3^
Global cognition				
MoCA-T	Low (T1)	Ref.	71/74	1.00
	Moderate (T2)	0.02 (−0.45, 0.50)	58/88	0.87 (0.50, 1.51)
	High (T3)	0.54 (0.06, 1.02)	48/97	0.54 (0.31, 0.95)
		*p_trend_*^4^ = 0.02		*p_trend_*^4^ = 0.03
Cognitive domains (composite Z score for each domain)				
Logical memory	Low (T1)	Ref.	55/90	1.00
	Moderate (T2)	0.08 (−0.08, 0.23)	42/104	1.13 (0.62, 2.07)
	High (T3)	0.09 (−0.06, 0.24)	48/97	0.76 (0.42, 1.38)
		*p_trend_*^4^ = 0.23		*p_trend_*^4^ = 0.38
Verbal fluency	Low (T1)	Ref.	55/90	1.00
	Moderate (T2)	−0.004 (−0.12, 0.11)	42/104	1.18 (0.69, 2.03)
	High (T3)	0.07 (−0.05, 0.19)	48/97	0.94 (0.53, 1.64)
		*p_trend_*^4^ = 0.23		*p_trend_*^4^ = 0.84
Executive function	Low (T1)	Ref.	43/101	1.00
	Moderate (T2)	−0.06 (−0.22, 0.10)	55/91	1.27 (0.75, 2.13)
	High (T3)	−0.07 (−0.23, 0.09)	46/97	1.12 (0.65, 1.91)
		*p_trend_*^4^ = 0.38		*p_trend_*^4^ =0.68
Attention	Low (T1)	Ref.	55/89	1.00
	Moderate (T2)	−0.008 (−0.13, 0.11)	57/89	1.03 (0.60, 1.76)
	High (T3)	0.12 (0.0003, 0.25)	38/105	0.56 (0.32, 0.99)
		*p_trend_*^4^ = 0.05		*p_trend_*^4^ = 0.049

^1^ Cognitive change was estimated by the regression coefficient (β) when diet quality increased one tertile. ^2^ Cognitive decline indicated the lowest tertile (T1) of the cognitive change over 2 years, and higher tertiles (T2 + T3) indicated normal cognition. ^3^ Multivariable models were adjusted for age, sex, years of education, *APOE* e4 status, the corresponding cognitive variable at baseline, total calories, depressive symptoms, quantity-adjusted vegetable variety score, and additional variables that remained significantly associated with the outcome variables in the model. Additional variables adjusted for different outcome variables included annual disposable income for global cognition and logical memory domain, and BMI for verbal fluency domain. ^4^ *p_trend_* indicated a linear trend across tertiles of diet quality. Abbreviations: mAHEI, modified Alternative Healthy Eating Index; T, tertile; AOR, adjusted odds ratio; CI, confidence interval; Ref., reference; MoCA-T, Montreal Cognitive Assessment—Taiwanese version; BMI, body mass index. Numbers in bold indicate significant findings (*p* < 0.05).

**Table 3:** Association between quantity-adjusted vegetable variety and cognitive decline over 2 years.

Cognitive Function	Quantity-Adjusted Vegetable Variety	β (95% CI) ^1,3^	N (Decliners/Non-Decliners)	AOR (95% CI) ^2,3^
Global cognition				
MoCA-T	Low (T1)	Ref.	56/93	1.00
	Moderate (T2)	−0.15 (−0.62, 0.32)	63/80	1.11 (0.64, 1.95)
	High (T3)	−0.07 (−0.54, 0.40)	58/86	0.87 (0.50, 1.52)
		*p_trend_*^4^ = 0.77		*p_trend_*^4^ = 0.61
Cognitive domains (composite Z score for each domain)				
Logical memory	Low (T1)	Ref.	42/107	1.00
	Moderate (T2)	0.02 (−0.13, 0.17)	49/94	1.04 (0.57, 1.91)
	High (T3)	−0.12 (−0.27, 0.03)	54/90	1.21 (0.67, 2.20)
		*p_trend_*^4^ = 0.12		*p_trend_*^4^ = 0.52
Verbal fluency	Low (T1)	Ref.	42/107	1.00
	Moderate (T2)	−0.04 (−0.16, 0.07)	49/94	1.34 (0.77, 2.33)
	High (T3)	−0.03 (−0.14, 0.09)	54/90	1.32 (0.76, 2.29)
		*p_trend_*^4^ = 0.67		*p_trend_*^4^ = 0.32
Executive function	Low (T1)	Ref.	55/91	1.00
	Moderate (T2)	0.10 (−0.06, 0.25)	46/97	0.73 (0.43, 1.22)
	High (T3)	0.08 (−0.08, 0.24)	43/101	0.62 (0.37, 1.04)
		*p_trend_*^4^ = 0.32		*p_trend_*^4^ = 0.07
Attention	Low (T1)	Ref.	55/91	1.00
	Moderate (T2)	0.08 (−0.04, 0.20)	46/97	0.82 (0.48, 1.42)
	High (T3)	0.08 (−0.04, 0.20)	49/95	0.79 (0.46, 1.36)
		*p_trend_*^4^ = 0.20		*p_trend_*^4^ = 0.39

^1^ Cognitive change was estimated by the regression coefficient (β) when quantity-adjusted vegetable variety increased one tertile. ^2^ Cognitive decline indicated the lowest tertile (T1) of the cognitive change over 2 years, and higher tertiles (T2 + T3) indicated normal cognition. ^3^ Multivariable models were adjusted for age, sex, years of education, *APOE* e4 status, the corresponding cognitive variable at baseline, total calories, depressive symptoms, mAHEI score, and additional variables that remained significantly associated with the outcome variables in the model. Additional variables adjusted for different outcome variables included annual disposable income for global cognition and logical memory domain, and BMI for verbal fluency domain. ^4^ *p_trend_* indicated a linear trend across tertiles of quantity-adjusted vegetable variety. Abbreviations: mAHEI, modified Alternative Healthy Eating Index; T, tertile; AOR, adjusted odds ratio; CI, confidence interval; Ref., reference; MoCA-T, Montreal Cognitive Assessment—Taiwanese version; BMI, body mass index. Numbers in bold indicate significant findings (*p* < 0.05).

## Supplementary Materials

The following are available online at https://www.mdpi.com/2072-6643/11/7/1666/s1. Table S1: Food items used for estimating the modified Alternative Healthy Eating Index (mAHEI), Table S2: Food components and scoring criteria of modified Alternative Healthy Eating Index (mAHEI), Table S3: The characteristics of the study population by tertiles of quantity-adjusted vegetable variety at baseline (2011–2013), Table S4: Association between diet quality and cognitive decline over 2 years stratified by important covariates, Table S5: Comparison of diet quality indexes construction criteria.
