# Supplementary material for: Association of Diet Quality and Vegetable Variety with the Risk of Cognitive Decline in Chinese Older Adults
Source: Nutrients. 2019 Jul 20;11(7):1666. doi: 10.3390/nu11071666 (PMC6682985; doi:10.3390/nu11071666)
Supplement: Supplementary file 1 [file nutrients-11-01666-s001.pdf]

nutrients

MDPI

Table S1. Food items used for estimating the modified Alternative Healthy Eating Index (mAHEI).

Food Components	Food Items
Fruits	Citrus, persimmon, papaya, mango, and other fruits
Vegetables	Tomato, tuber, carrot and pumpkin, light-colored vegetable, spinach and broccoli, and other dark-green vegetables
Soy protein	Soymilk and soybean products (e.g., tofu or dried tofu)
Fish	Deep-sea fish, other fish, and seafood
Meat	Beef, pork, lamb, poultry, offal food
Eggs	Chicken's egg and duck's egg
Whole grain	Whole wheat bread, brown rice, and germ rice
Fried foods	Fried food
Alcohol	Alcohol (concentration: <10%, 10%–19%, 20%–39%, 40%–50% and >50%)

One drink is defined as 12 fluid ounces of regular beer (5% alcohol), 5 fluid ounces of wine (12% alcohol), or 1.5 fluid ounces of 80 proof (40% alcohol) distilled spirits. One drink contains 0.6 fluid ounces of alcohol (14 g).

Table S2. Food components and scoring criteria of modified Alternative Healthy Eating Index (mAHEI).

Food Components	Minimum Score of 0	Maximum Score of 10
Fruits (servings/day)	0	4
Vegetables (servings/day)	0	5
Soy protein (servings/day)[1]	0	1
Fish/(meat + eggs)	0	4
Whole grain (servings/day)	0	≥3
Fried foods (servings/day)	≥4	≤0.5
Alcohol (servings/day)	Men: 0 or >3.5<br>Women: 0 or >2.5	Men: 1.5–2.5<br>Women: 0.5–1.5
Total score	0	70

Score of each food component ranged from 0 to 10.

[1] The Modified Alternative Healthy Eating Index was adapted from Dehghan et al., 2012 [33] with minor revision on the component "nuts and soy protein". That is, nuts were excluded from the component of "nuts and soy protein" because nuts are not commonly consumed by Chinese older adults.

Table S3. The characteristics of the study population by tertiles of quantity-adjusted vegetable variety at baseline (2011 – 2013).

Variables	Quantity-Adjusted Vegetable Variety				*p*-value
	Overall (n = 436)	T1 (n = 149)	T2 (n = 143)	T3 (n = 144)	
	Mean, SD				
Vegetable variety[1]	3.4, 1.0	**2.5, 0.9**	**3.4, 0.5**	**4.3, 0.5**	**<0.0001**
mAHEI score	36.5, 7.6	36.0, 7.8	36.4, 7.7	37.2, 7.5	0.37
Age (years)	72.5, 5.2	72.3, 5.0	72.5, 5.4	72.6, 5.3	0.86
Years of education (years)	14.0, 3.4	13.9, 3.3	14.1, 3.4	14.0, 3.6	0.87
BMI (kg/m^2)	23.5, 2.6	23.5, 2.6	23.8, 3.3	24.3, 2.8	0.10
Physical activity (MET-min/week)	1747.5, 1444.0	1757.7, 1430.4	1771.1, 1517.6	1713.6, 1391.1	0.94
Gait speed (m/s)	0.8, 0.2	0.9, 0.2	0.8, 0.2	0.8, 0.3	0.21
Total energy intake	1683.0, 395.1	1661.0, 413.3	1714.4, 384.4	1674.5, 387.0	0.49

(kcal/day)					
MoCA-T	27.0, 4.6	27.0, 2.1	27.0, 2.1	27.2, 2.2	0.64
			**N (%)**		
Women	231 (53)	76 (51)	74 (52)	81 (56)	0.63
Annual disposable income (TWD per year)					**0.002**
<300K	68 (16)	**11 (8)**	**26 (19)**	**31 (22)**	
300-800K	106 (26)	**32 (23)**	**35 (26)**	**39 (28)**	
800–1000K	61 (15)	**18 (13)**	**20 (15)**	**23 (17)**	
>1000K	177 (43)	**79 (56)**	**53 (40)**	**45 (33)**	
Supplement use	346 (79)	119 (80)	105 (73)	122 (85)	0.06
Depressive symptoms[2]	43 (10)	12 (8)	13 (9)	18 (13)	0.41
Hypertension	280 (65)	88 (60)	93 (66)	99 (70)	0.24
Diabetes mellitus	68 (16)	26 (17)	26 (18)	16 (11)	0.19
APOE e4 carriers	67 (16)	20 (14)	28 (20)	19 (13)	0.21

[1] Vegetable variety was defined as the number of five vegetable groups consumed at least one cup equivalent per week (score range: 0–5). [2] Depressive symptoms (yes/no) were defined as at least one of the following three factors: CES-D scores ≥ 16, use of antidepressants, or self-report diagnosis of depression. Abbreviations: T, tertile; mAHEI, modified Alternative Healthy Eating Index; SD, standard deviation; BMI, body mass index; MET, metabolic equivalent of task; MoCA-T, Montreal Cognitive Assessment—Taiwanese version; TWD, Taiwan dollar; *APOE*, apolipoprotein E; CES-D, Center for Epidemiologic Studies Depression Scale.
Numbers in bold indicate significant findings ($p < 0.05$).

Table S4. Association between diet quality and cognitive decline over 2 years stratified by important covariates.

Cognitive Function	Diet Quality (mAHEI)	Linear Regression[1, 3]			Logistic Regression[2, 3]				
		β (95% CI)	β (95% CI)	$p_{interaction}$	N (Decliners/ Non-Decliners)	AOR (95% CI)[2]	N (Decliners/ Non-Decliners)	AOR (95% CI)[2]	$p_{interaction}$
**MoCA-T**		**Low Vegetable Variety[4]**	**High Vegetable Variety[4]**		**Low Vegetable Variety[4]**		**High Vegetable Variety[4]**		
	**Low**	Ref.	Ref.	0.84	23/25	1.00	48/49	1.00	0.90
	**Moderate**	-0.16 (-0.92, 0.60)	0.07 (-0.57, 0.70)		22/40	0.91 (0.34, 2.50)	36/48	0.89 (0.45, 1.78)	
	**High**	0.22 (-0.60, 1.03)	0.59 (-0.01, 1.19)		11/28	0.84 (0.27, 2.58)	37/69	**0.49 (0.26, 0.95)**	
		p_{trend} = 0.58	p_{trend} = 0.05			p_{trend} = 0.75		p_{trend} = **0.03**	
		**Age 65–74 (years)**	**Age ≥ 75 (years)**		**Age 65–74 (years)**		**Age ≥ 75 (years)**		
	**Low**	Ref.	Ref.	0.37	45/55	1.00	26/19	1.00	0.20
	**Moderate**	-0.08 (-0.56, 0.39)	0.57 (-0.73, 1.88)		46/70	0.91 (0.47, 1.77)	12/18	0.43 (0.13, 1.37)	
	**High**	0.46 (-0.04, 0.97)	0.92 (-0.22, 2.05)		24/67	**0.42 (0.20, 0.88)**	24/30	0.56 (0.21, 1.50)	
		p_{trend} = 0.07	p_{trend} = 0.11			p_{trend} = **0.02**		p_{trend} = 0.27	
		**Men**	**Women**		**Men**		**Women**		
	**Low**	Ref.	Ref.	0.93	36/34	1.00	35/40	1.00	0.78
	**Moderate**	-0.14 (-0.91, 0.63)	0.14 (-0.47, 0.74)		25/40	0.82 (0.36, 1.86)	33/48	0.96 (0.43, 2.11)	
	**High**	0.49 (-0.26, 1.23)	0.52 (-0.11, 1.14)		25/45	0.54 (0.24, 1.20)	23/52	0.64 (0.28, 1.47)	
		p_{trend} = 0.20	p_{trend} = 0.10			p_{trend} = 0.13		p_{trend} = 0.29	
		***APOE* e4 Non-Carriers**	***APOE* e4 Carriers**		***APOE* e4 Non-Carriers**		***APOE* e4 Carriers**		
	**Low**	Ref.	Ref.	**0.05**	60/62	1.00	9/11	1.00	0.35
	**Moderate**	-0.03 (-0.54, 0.48)	0.19 (-1.18, 1.57)		51/70	0.93 (0.51, 1.68)	6/18	0.47 (0.09, 2.45)	
	**High**	**0.74 (0.23, 1.26)**	-0.29 (-1.66, 1.08)		38/82	**0.49 (0.27, 0.91)**	9/14	1.02 (0.21, 4.94)	
		p_{trend} = **0.005**	p_{trend} = 0.68			p_{trend} = **0.02**		p_{trend} = 0.95	
**Attention**		**Low Vegetable Variety[4]**	**High Vegetable Variety[4]**		**Low Vegetable Variety[4]**		**High Vegetable Variety[4]**		

**Low**	Ref.	Ref.	0.10	22/25	1.00	33/64	1.00	0.44
**Moderate**	0.10 (-0.10, 0.30)	-0.08 (-0.24, 0.07)		23/39	0.71 (0.28, 1.81)	34/50	1.33 (0.67, 2.63)	
**High**	0.07 (-0.16, 0.29)	0.15 (-0.001, 0.30)		10/27	0.51 (0.17, 1.52)	28/78	0.58 (0.29, 1.14)	
	p_{trend} = 0.54	p_{trend} = 0.05			p_{trend} = 0.23		p_{trend} = 0.11	
	**Age 65–74 (years)**	**Age ≥ 75 (years)**		**Age 65–74 (years)**		**Age ≥ 75 (years)**		
**Low**	Ref.	Ref.	0.70	37/62	1.00	18/27	1.00	0.06
**Moderate**	-0.03 (-0.16, 0.11)	0.03 (-0.24, 0.30)		49/67	1.49 (0.77, 2.89)	8/22	0.40 (0.13, 1.22)	
**High**	0.08 (-0.07, 0.23)	0.18 (-0.06, 0.42)		25/65	0.71 (0.34, 1.46)	13/40	**0.35 (0.13, 0.95)**	
	p_{trend} = 0.30	p_{trend} = 0.13			p_{trend} = 0.37		p_{trend} = **0.04**	
	**Men**	**Women**		**Men**		**Women**		
**Low**	Ref.	Ref.	0.16	23/46	1.00	32/43	1.00	0.14
**Moderate**	0.12 (-0.07, 0.30)	-0.11 (-0.28, 0.06)		18/47	0.74 (0.31, 1.72)	39/42	1.27 (0.61, 2.65)	
**High**	0.16 (-0.02, 0.34)	0.12 (-0.06, 0.30)		21/49	0.70 (0.30, 1.63)	17/56	**0.36 (0.16, 0.83)**	
	p_{trend} = 0.09	p_{trend} = 0.22			p_{trend} = 0.41		p_{trend} = **0.02**	
	***APOE* e4 Non-Carriers**	***APOE* e4 Carriers**		***APOE* e4 Non-Carriers**		***APOE* e4 Carriers**		
**Low**	Ref.	Ref.	0.06	44/78	1.00	10/9	1.00	0.68
**Moderate**	-0.08 (-0.21, 0.06)	**0.35 (0.03, 0.67)**		50/71	1.14 (0.63, 2.04)	7/17	0.65 (0.13, 3.25)	
**High**	0.09 (-0.05, 0.23)	0.31 (-0.02, 0.64)		33/85	0.60 (0.32, 1.11)	5/18	0.32 (0.06, 1.67)	
	p_{trend} = 0.21	p_{trend} = 0.06			p_{trend} = 0.11		p_{trend} = 0.18	

[1] Cognitive change was estimated by the regression coefficient (β) when diet quality increased per tertile. [2] Cognitive decline indicated the lowest tertile (T1) of the cognitive change over 2 years, and higher tertiles (T2 + T3) indicated normal cognition. [3] Multivariable models were adjusted for age, sex, years of education, *APOE* e4 status, the corresponding cognitive variable at baseline, total calories, depressive symptoms, and quantity-adjusted vegetable variety. Additional variables adjusted for different outcome variables included annual disposable income for global cognition. [4] Low vegetable variety was defined as the lowest tertile (T1) of the quantity-adjusted vegetable variety, and high vegetable variety was defined as the remaining tertiles (T2 + T3). Abbreviations: mAHEI, modified Alternative Healthy Eating Index; T, tertile; AOR, adjusted odds ratio; CI, confidence interval; MoCA-T, Montreal Cognitive Assessment—Taiwanese version; *APOE*, apolipoprotein E. Numbers in bold indicate significant findings ($p_{trend} < 0.05$ and $p_{interaction} < 0.10$).

Table S5. Comparison of diet quality indexes construction criteria.

Index	mAHEI	AHEI-2010	HEI-2005	C-HEI	HDI
**Dimensions**	**Food or Nutrient Indicator (Weighted Score)**				
**Adequacy**[1]	Vegetables (10)	Vegetables (10)	Total vegetables (5)	Fruits and vegetables (0–20)	Fruits and vegetables (1)
			Dark-green, orange vegetables and legumes (5)		
	Fruits (10)	Fruits (10)	Total fruits (5)		
			Whole fruits (5)		
	Nuts and soy protein (10)	Nuts and legumes (10)	Beans and meats (10)	Meat and meat alternatives (10)	
	Ratio of fish/(meat + eggs) (10)				Fish (1)
	Whole grains (10)	Whole grains (10)	Total grains (5)<br>Whole grains (5)	Grains (10)	
		PUFA (10)	Oil (10)[3]		
		Long-chain n-3 fats (10)	Milk (10)	Milk (10)	Fiber (1)
**Moderation**[2]	Deep-fried food (10)	Trans fat (10)		Total fat (10)	PUFA (1)
		Red or processed meat (10)	Saturated fat (10)	Saturated fat (10)	Saturated fat (1)
				Cholesterol (10)	Cholesterol (1)
	Alcohol (10)	Alcohol (10)	Calories from solid fats, alcohol, and added sugar (20)		Total carbohydrates (1)
		Sugar-sweetened beverages and fruit juice (10)			Sucrose (-1)
		Sodium (10)	Sodium (10)	Sodium (10)	
					Protein (1)
**Variety**				Dietary variety (10)[4]	
**Total score**	70	110	100	100	8

[1]Adequacy indicated the sufficient intake of dietary elements beneficial to health. [2]Moderation indicated limiting dietary elements harmful to health. [3]Oils include nonhydrogenated vegetable oils and oils in fish, nuts, and seed. [4]Dietary variety as daily consumption of at least one portion from each of four food groups (vegetables, fruits, whole grains, and protein foods). Abbreviations: PUFA, polyunsaturated fatty acid; mAHEI, modified Alternative Healthy Eating Index; AHEI, Alternative Healthy Eating Index; HEI, Healthy Eating Index, C-HEI, Canadian Healthy Eating Index; HDI, Healthy Diet Indicator.
